# Structural Characterization and In Vitro Fermentation Properties of Polysaccharides from *Polygonatum filipes*

**DOI:** 10.3390/foods15091561

**Published:** 2026-05-01

**Authors:** Huimin Hu, Jiawei Wang, Kaijun Wang, Ke Chen, Nike Ding, Fenghua Wu, Guanyu Fang, Xingquan Liu, Chaojun Ye, Peng Wang

**Affiliations:** 1 College of Food and Health, Zhejiang Agriculture and Forestry University, Hangzhou 311300, China; 2 National Grain Industry (High-Quality Rice Storage in Temperate and Humid Region) Technology Innovation Center, Zhejiang Agriculture and Forestry University, Hangzhou 311300, China; 3 Key Laboratory of Horticultural Plant Breeding, Wenzhou Vocational College of Science and Technology, Wenzhou Academy of Agricultural Sciences, Wenzhou 325000, China

**Keywords:** *Polygonatum filipes*, polysaccharides, SCFAs, in vitro fermentation

## Abstract

In this study, a homogeneous polysaccharide, designated as PFP-80, was isolated from the dried root of *Polygonatum filipes* using enzymatic extraction combined with graded ethanol precipitation. Structural characterization suggested that PFP-80 was a fructan polysaccharide with a molecular weight of 4.06 kDa. The analysis with gas chromatograph–mass spectrometer (GC–MS) and nuclear magnetic resonance (NMR) further confirmed that PFP-80 consisted of →1)-β-D-*Fruf*-(2→ and →1,6)-β-D-Fru*f*-(2→ linkages, with branching occurring at the *O*-6 position. After 48 h of fermentation, the pH was decreased while SCFAs were increased significantly due to the utilization of PFP-80. Furthermore, PFP-80 was found to modulate the gut microbiota by enhancing microbial abundance and diversity, and by impeding the growth of deleterious pathogens such as *Ruminococcus gnavus*. In summary, the present results provide a scientific basis for the subsequent development of PFP-derived functional food products.

## 1. Introduction

*Polygonatum filipes*, a perennial herbaceous plant, has been cultivated all around China, mainly in the Zhejiang, Anhui, and Fujian provinces, and typically grows in forests, shrublands, or grassy slopes at elevations of 200–600 m. *Polygonatum filipes* has been used as both food and a traditional medicinal herb with a long history of use in China. It is traditionally employed to strengthen the spleen, moisten the lungs, invigorate qi, and nourish yin [1]. Recent studies have reported that *Polygonatum* species contain polysaccharides, saponins, polyphenols, flavonoids, and other bioactive compounds with various functional properties [2]. They exhibited diverse biological activities, including hypoglycemic, immunomodulatory, and improvement of learning memory, etc. refs. [2,3,4].

Among plant-based compounds, polysaccharides are regarded as one of the main bioactive components. Polysaccharides from *Polygonatum* species exhibit various functional activities, including the capacities of lowering blood glucose, reducing blood lipids, and anti-cancer, anti-inflammation, anti-bacteria, and immune regulation [5], with potential applications in functional foods, nutraceuticals, and the pharmaceutical industry [6,7]. Most previous studies on *Polygonatum* polysaccharides have focused on species such as *Polygonatum sibiricum*, *Polygonatum cyrtonema Hua*, and *Polygonatum kingianum*, in which fructan-type polysaccharides were commonly reported. However, although these species belong to the same genus, differences in plant origin may lead to changes in polysaccharide structure, such as molecular weight, monosaccharide composition, and glycosidic linkage patterns, which may further influence their biological functions [8,9]. In contrast, little attention has been given to *Polygonatum filipes*, and its polysaccharides have not been well studied, especially in terms of their detailed structures. However, the bioactivities of polysaccharides are largely dependent on their structural characteristics. Since the bioactivities of polysaccharides are dependent on their structural characteristics, the separation and purification of crude polysaccharides to obtain homogeneous fractions are essential for resolving the structural properties and evaluating biological activity.

In recent years, increasing attention has been directed toward the extraction of natural polysaccharides for their potential to regulate intestinal microbiota homeostasis. However, their high molecular weight and complex structures caused them to be resistant to digestion in the upper gastrointestinal tract, which lacks sufficient carbohydrate-active enzymes (CAZymes). Many polysaccharides reach the colon intact, where they are degraded into monosaccharides by CAZymes produced by the colonic microbiota. These monosaccharides are further utilized by gut microbiota, leading to the production of SCFAs and a reduction in intestinal pH levels. The acidic condition established in the gut exerted a suppressive effect on pathogenic bacterial growth, thereby promoting intestinal health [8]. Adlay seed bran polysaccharides (ASBP) have been shown to modulate gut microbiota by increasing beneficial Bacteroidetes, inhibiting Proteobacteria, and enhancing SCFA production, with ASBP-CF showing the highest fermentability and prebiotic activity [9]. Similarly, jujube polysaccharides acted as prebiotics by stimulating beneficial bacteria and suppressing harmful species, while soybean polysaccharides promoted the proliferation of probiotics and contributed to microbiota homeostasis [10]. Overall, the prebiotic potential of the polysaccharides has emerged as a key focus in gut microbiome research.

In this study, it was hypothesized that PFP-80 could be used as a carbon source, degraded, and utilized during in vitro fermentation, thereby exhibiting potential probiotic properties. A pure polysaccharide fraction (PFP-80) was extracted from *Polygonatum filipes* using a combination of enzymatic hydrolysis and graded ethanol precipitation. Its chemical structure was characterized through monosaccharide composition, FTIR, methylation analysis, and NMR. Based on these structural analyses, in vitro fermentation models were used to investigate changes in the physicochemical characteristics of PFP-80 during fermentation. A 16S rRNA gene sequencing technology was applied to monitor alterations in the composition and relative abundance of the gut microbiota throughout the fermentation process. This study aimed to evaluate the probiotic potential of PFP-80 and provide a theoretical basis for its development as a functional food or dietary supplement.

## 2. Materials and Methods

### 2.1. Materials

#### 2.1.1. Chemicals and Reagents

*Polygonatum filipes* (PF) was obtained from Taizhou, Zhejiang province, China. The thermostable α-amylase (20,000 U/mL) and glucoamylase (100,000 U/g) were obtained from Aladdin Chemistry Co., Ltd. (Shanghai, China). Compound proteinase (100,000 U/g) was obtained from Solarbio (Beijing, China). Standard samples of SCFAs were purchased from Aladdin Chemistry Co., Ltd. (Shanghai, China). The other chemicals (such as inulin, hemoglobin, vitamin K, and bladed azulene) were obtained from Yuanye Bio-Technology Co., Ltd. (Shanghai, China).

#### 2.1.2. Purification and Extraction Procedures for PFP

PF was dried at 70 °C until a constant weight, ground into powder (60 mesh), and soaked in 90% ethanol for 24 h to remove lipids. The filtered residue was dried, mixed with deionized water (1:25, *w*/*v*), and treated with 2% thermostable α-amylase at 90 °C and pH 6.0 for 2 h. The extract was cooled and sequentially hydrolyzed with 1.5% glucoamylase and 1.5% compound proteinase for 2 h. The solution was heated to 100 °C for 10 min to inactivate enzymes, adjusted to pH 4.0, and centrifuged to collect the supernatant. Then, ethanol (95%, *v*/*v*) was added to the supernatant to reach final concentrations of 20%, 40%, 60%, and 80% (*v*/*v*), respectively. The mixture was stored at 4 °C for 12 h and then centrifuged to collect the precipitates. The precipitates were dialyzed (Mw 3500 Da) for 60 h before freeze-drying.

### 2.2. Characterization of Chemical Composition

The phenol-sulfuric acid assay was applied to quantify total polysaccharide content, with anhydrous glucose as the standard. The uronic acid content was assayed via the carbazole-sulfuric acid method, with galacturonic acid as the standard. The protein content was measured using the Coomassie brilliant blue assay. All samples were analyzed in triplicate.

### 2.3. Structural Characterization

#### 2.3.1. Determination of Molecular Weight

A total of 3 mg of PFP was dissolved in 1.5 mL of 0.02 M KH_2_PO_4_ and then filtered through a 0.22 μm membrane prior to HPLC analysis using a C18 column (5 μm, 4.6 mm × 250 mm). The flow rate was set to 0.6 mL/min, and the column temperature was maintained at 35 °C. The molecular weight of PFP was estimated by comparing its retention time with those of molecular weight standards (Mw 5000, Mw 12,000, Mw 25,000, Mw 50,000, and Mw 410,000).

#### 2.3.2. Monosaccharide Composition Analysis

The 5 mg of PFP-80 was hydrolyzed with 3 M trifluoroacetic acid (TFA) at 80 °C for 2 h. The hydrolysates were evaporated to dryness under a nitrogen stream. Chromatographic analysis of the dried residue was carried out using a Thermo ICS5000+ ion chromatography system (Thermo Fisher Scientific, Waltham, MA, USA), fitted with a Dionex Carbopac^TM^ PA20 column (150 mm× 3.0 mm, 6 μm). Mobile phases: A (H_2_O), B (15 mM NaOH), and C (15 mM NaOH + 100 mM NaAC). Elution gradient (*v*/*v*, min: A/B/C): 0–18 min: 98.8:1.2:0; 18–20 min: 98.8:1.2:0 → 50:50:0; 20–30 min: 50:50:0; 30.1–46 min: 0:0:100; 46.1–50 min: 0:100:0; and 50.1–80 min: 98.8:1.2:0.

#### 2.3.3. FTIR Spectra

A mixture of PFP and KBr (1:100, *w*/*w*) was ground into powder and compressed into a pellet. The prepared pellet was subsequently loaded into the FTIR spectrometer, with spectral measurements performed across a wavenumber range of 4000–400 cm^−1^.

#### 2.3.4. Methylation

The methylation analysis of PFP-80 was conducted by Jiangsu Sanshu Biotechnology Co., Ltd. (Nantong, China). PFP-80 (6.0 mg) was dissolved in 1 mL of dimethyl sulfoxide, and then 50 mg of NaOH powder and 1.0 mL of CH_3_I were added to the solution. Maintained in an ice bath, the mixture was shielded from light. Subsequently, the prepared solution was hydrolyzed with 0.2 mL of 2 M TFA at 60 °C for 1 h. A total of 2.0 mL of ultrapure water, 100 μL of 2 M NH_3_, and 100 μL of NaBD_4_ were sequentially added. The mixture was subsequently acetylated with 3.0 mL of acetic anhydride. The partially methylated alditol acetates were analyzed by GC-MS analysis using an Agilent 7890A-5977B system (Agilent Technologies, Santa Clara, CA, USA) equipped with a BPX70 column (30 m × 0.25 mm × 0.25 μm).

#### 2.3.5. NMR Spectroscopy

A total of 30 mg of the PFP-80 was added to D_2_O to obtain the solution. At 25 °C, 1D-NMR spectra (1 H-NMR, 13 C-NMR) and 2D-NMR spectra (COSY, HSQC, HMBC, and NOESY) were collected by means of a Bruker AVANCE NEO 500 MHz nuclear magnetic resonance spectrometer manufactured by Bruker in Germany.

### 2.4. In Vitro Fermentation of PFP-80 by Human Fecal Microbiota

In vitro fecal fermentation of PFP-80 was carried out on the basis of methods described earlier, with slight modifications [11,12]. The fermentation medium was prepared and adjusted to pH 7.0, followed by the addition of PFP-80 at a final concentration of 5 mg/mL. Fecal samples were collected from four healthy volunteers (two males and two females, aged 22–26 years) who had not used antibiotics within the past three months. All donors were non-smokers and had no known gastrointestinal diseases. To minimize variability, the donors generally maintained similar dietary habits and living environments during the sampling period. A total of 5 g of feces was taken from each tube and added to PBS buffer containing 0.1% L-cysteine hydrochloride to prepare a fecal suspension (10%, *w*/*v*) under anaerobic conditions. After the mixture was thoroughly mixed for 5 min, a human fecal inoculum was obtained. Equal amounts of each fecal sample were homogenized in 0.1 M PBS (10%, *w*/*v*) and subsequently filtered through sterile gauze to obtain a uniform fecal slurry. The fecal slurry was then added to the fermentation medium containing PFP-80 and incubated in an anaerobic chamber at 37 °C for 48 h. A group without PFP-80 served as a blank control (BLK), and a group with inulin was used as a positive control (INL). Finally, samples were collected at 6 h intervals and centrifuged at 12,000 *g* for 10 min to separate the supernatant and fecal pellets. All samples were stored at −80 °C until further analysis. Three replicates of each sample were measured.

### 2.5. Quantification of Short-Chain Fatty Acids (SCFAs) and pH Values

During in vitro fecal fermentation, pH values were tracked at regular intervals with a pre-calibrated meter. The SCFA contents were quantified by GC-MS using a QP2010-plus system (Shimadzu Co., Ltd., Kyoto, Japan) equipped with an Rxi-5 MS column (0.25 μm, 30.0 m × 0.25 mm). A 1 μL injection volume was used with helium as the carrier gas at 0.8 mL/min. The detector and inlet temperatures were kept at 240 °C, while the oven was held at 150 °C for 1 min before being enhanced to 250 °C at 5 °C min^−1^. SCFA concentrations were calculated using standard curves. The limits of detection (LOD) and quantification (LOQ) were determined based on signal-to-noise ratios (S/N) of 3 and 10, respectively, using low-concentration standard solutions. The LODs ranged from 0.01 to 0.05 mM, while the LOQs ranged from 0.03 to 0.15 mM.

### 2.6. Analysis of Gut Microbiota Composition

The genomic DNA was isolated from fecal fermentation samples collected at different times using the fecal genomic DNA extraction kit (DP812, Tiangen Biotech, Beijing, China), and preserved at −20 °C for follow-up analysis.

16S V3-V4 amplicons (338F/806R) were purified (Monarch^®^ kit, NEB, Ipswich, MA, USA) and sequenced on Illumina NovaSeq 6000 (Illumina, San Diego, CA, USA). The raw sequencing data were processed using the DADA2 plugin in QIIME2 (version 2020.6). This process included quality filtering, removing errors, merging reads, and removing chimeras, which produced amplicon sequence variants (ASVs). The data were processed using the cloud platform provided by Beijing Baimaike Biotechnology Co., Ltd. (Beijing, China)

### 2.7. Statistical Analysis

Each experiment was conducted in triplicate, with data expressed as mean ± standard deviation (SD). Changes in pH and SCFA concentrations were analyzed using one-way analysis of variance (ANOVA) followed by Tukey’s post hoc test to evaluate differences between groups. Data visualization was performed using Origin 2021 software. Differences were considered statistically significant at *p* < 0.05.

## 3. Results and Discussion

### 3.1. Yield and Chemical Composition Determination

The polysaccharide fractions were obtained using the gradient ethanol precipitation method, and the yield of the PFP-80 was 5.49%. This was caused by a decrease in solvent polarity as the ethanol concentration increased. As a result, polysaccharides, which were polar molecules, were gradually precipitated based on the principle of “like dissolves like”, and the polysaccharide yield was increased [13]. Table 1 summarizes the chemical composition of the PFP. The total sugar contents of the PFP groups were significantly enhanced from 84.14% to 94.95% as the final ethanol concentration increased to 80% (*v*/*v*), suggesting that the PFP combined with graded ethanol precipitation could get high-purity polysaccharides. Additionally, the uronic acid contents of PFP and PFP-80 were 3.68% and 2.08%, respectively, indicating that these four PFP fractions were neutral polysaccharides. The protein contents in the PFP fractions ranged from 0.32% to 0.33%. This low protein content suggested that most of the protein was effectively removed from the polysaccharide.

### 3.2. Molecular Weight of PFP-80

**Figure 1:**
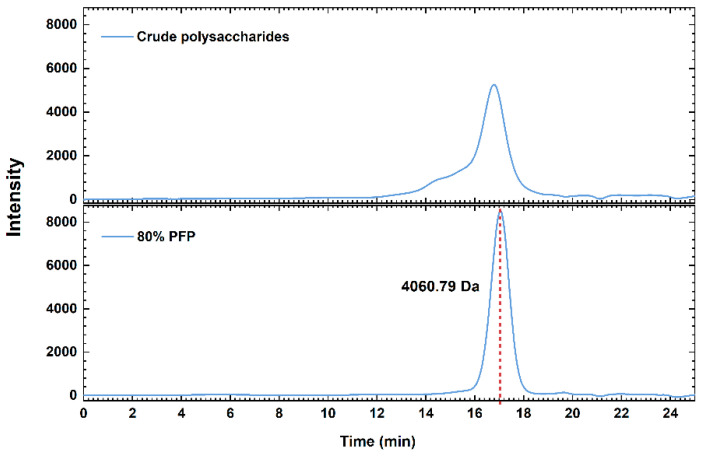
The GPC of PFP and PFP-80.

The molecular weight distribution chromatograms are shown in Figure 1. The PFP-80 showed a single symmetrical peak, which confirmed its identity as a homogeneous polysaccharide. According to the standard curve (Log M = −0.5758x + 13.426, R^2^ = 0.9972), the average molecular weight of PFP-80 was measured as approximately 4.060 kDa. The PFP-80 exhibited a lower molecular weight compared to the other groups. It was reported that higher-molecular-weight polysaccharides exhibited lower polarity and precipitated at lower ethanol concentrations, whereas low-molecular-weight polysaccharides were more water-soluble and required higher ethanol concentrations for precipitation [14].

### 3.3. Monosaccharide Composition of PFP-80

**Figure 2:**
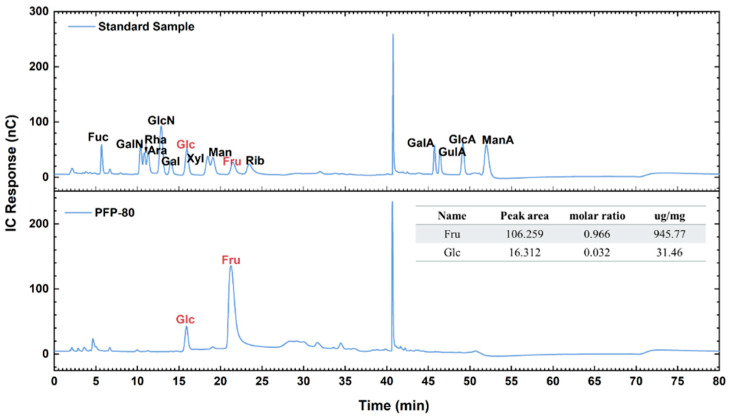
Monosaccharide composition analysis of PFP-80.

The monosaccharide composition of PFP-80 is shown in Figure 2. The molar ratio of fructose (Fru) to glucose (Glc) in PFP-80 was determined to be 96.6:3.2, suggesting that the polysaccharides might be classified as fructans. In addition, the monosaccharide compositions of PFP were different from those in the previous study. Cheng et al. [15] reported that *Polygomatum cyrtonema* polysaccharides (PCPs) were extracted by graded alcoholic precipitation, which was composed of Fru, Ara, Gal, Glu, Man, and Fru. The variations in monosaccharide composition might be attributed to the differences among *Polygonatum* species.

### 3.4. FTIR Spectra Analysis

**Figure 3:**
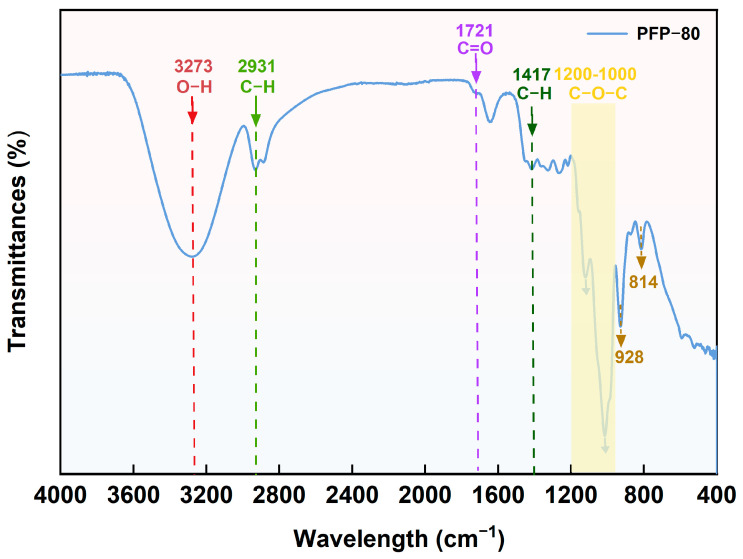
FTIR spectra of PFP-80 fractions.

The FTIR spectra of PFP-80 are shown in Figure 3. The O-H stretching vibration was attributed to the peak at 3273 cm^−1^. The peak detected at 2931 cm^−1^ was assigned to C-H stretching and bending vibrations, including those of CH, CH_2_, and CH_3_ groups [15]. The peaks at approximately 1417 cm^−1^ were attributed to the vibration of C-H. The peak at 1721 cm^−1^ was due to the C=O stretching vibrations, while the peak at 1647 cm^−1^ was related to the binding of water [16,17]. The absorption peaks in the range of 1200–1000 cm^−1^ were attributed to the stretching vibrations of the C-O-H bond and C-O bonds. Additionally, the absorption peaks located at 1120 cm^−1^ and 1015 cm^−1^ are assigned to the stretching vibrations of glycosidic C-O-C bonds within pyranose rings [11,18]. The characteristic absorption peaks observed at 928 cm^−1^, 814 cm^−1^, and 931 cm^−1^ indicated α-type and β-type glycosidic linkages, as well as the symmetric stretching vibration of the furan ring, respectively. These results suggested that PFP-80 was a furan-type glycosaminoglycan containing both α- and β-type polysaccharides [19].

### 3.5. Methylation Analysis

Methylation analysis was used to determine the linkage positions of monosaccharide residues. It was a common technique for investigating the higher-order structures of polysaccharides [20]. The results are shown in Table 2. PFP-80 consisted of four main types of glycosidic bonds, including Fru*f*-(2→, →1)-Fru*f*-(2→, 1,6)-Fru*f*-(2→, and →6)-Glc*p*-(1→. The fructose in PFP-80, which was the predominant monosaccharide, was primarily present in the forms of →1)-Fru*f*-(2→ of 54.59% and Fru*f*-(2→ of 21.87%. In addition, →6)-Glc*p*-(1→ (3.26%) was detected, which might be located at the branching sites or reducing ends of the polysaccharide.

### 3.6. NMR Analysis

**Figure 4:**
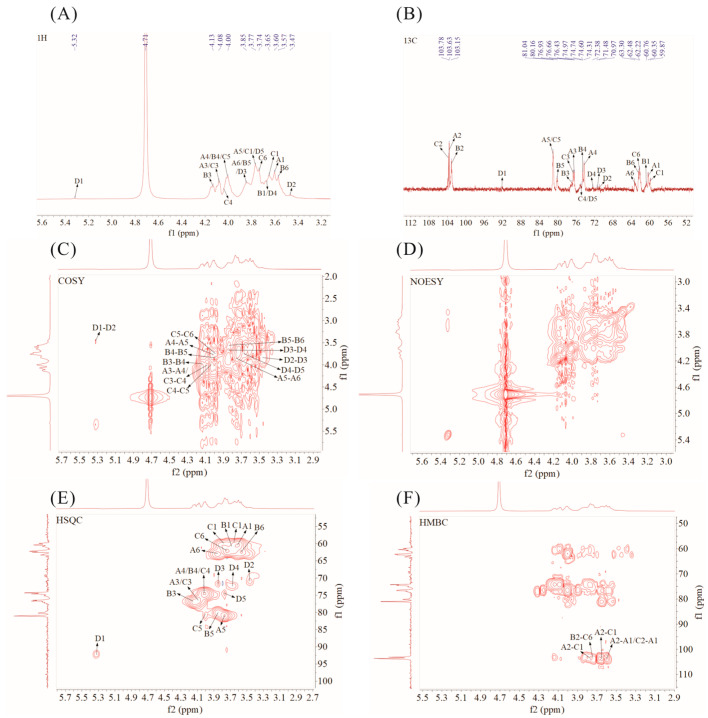
The ^1^H NMR (**A**),13C NMR (**B**), COSY (**C**), NOESY (**D**), HSQC (**E**), HMBC, and (**F**) spectra analysis of PFP-80.

NMR is widely employed to elucidate the structural characteristics of polysaccharides, such as the types of anomeric carbon and the order of linkage sites of the glycosidic bond. The results of NMR analysis of PFP-80 are shown in Figure 4. In the ^1^H NMR spectrum of PFP-80 (Figure 4A), many proton resonance signals were observed in the δ 3.0–5.5 region, representing a typical distribution diagram of the NMR signals of polysaccharides. There is a correlation between the glycosidic bond conformation and the chemical shifts in the anomer hydrogen, with the anomer hydrogen signals of the β-glycosidic bond conformation occurring within δ 4.3–4.8, while those of the α-glycosidic bond conformation are predominantly distributed in the range of δ 4.8 to 5.8. In the ^13^C NMR spectrum (Figure 4B), multiple signals corresponding to anomeric carbons were detected in the range of δ 90–110 [21]. In the HSQC spectrum (Figure 4E), no cross-peaks were detected in the isomer region, and anomeric proton signals corresponding to the ketose residues A, B, and C were absent, suggesting that these residues A, B, and C might be fructose residues. The chemical shifts in residue D were attributed to the HSQC spectra, suggesting that the sugar residue D might be →6)-α-D-Glc*p*-(1→ [22]. Combined analysis of NMR spectra and methylation data indicated that PFP-80 possessed a main chain primarily composed of →1)-β-D-Fru*f*-(2→ and →1,6)-β-D-Fru*f*-(2→, and bore branched chains consisting of β-D-Fru*f*-(2→ residues attached to the O-6 position of →1,6)-β-D-Fru*f*-(2→. Detailed chemical shift assignments and correlation signals are shown in Table S3. Based on this information, the putative structure of PFP-80 was drawn as shown in Figure S5.

### 3.7. Influences of PFP-80 on pH Values During the Fermentation Process

**Figure 5:**
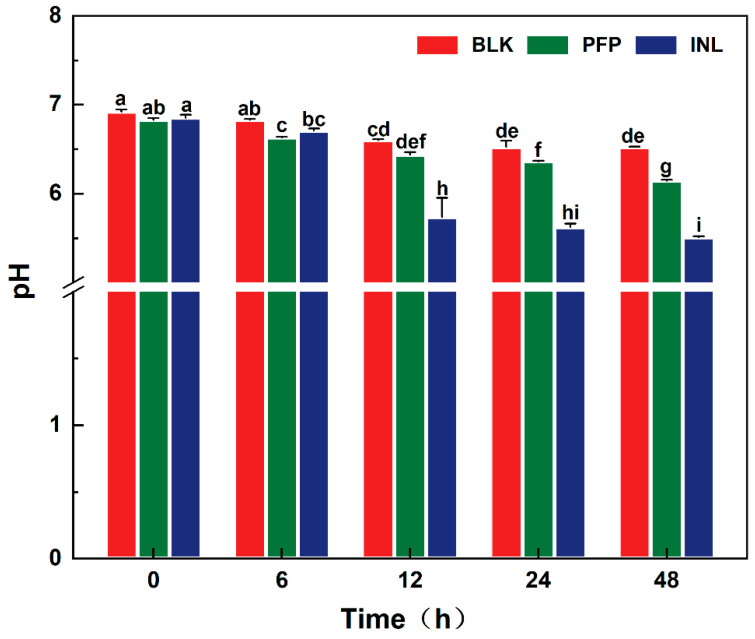
Changes in pH value during fermentation. Different letters indicated significant differences between groups (*p* < 0.05).

During this process, PFP-80 was metabolized by gut microbiota, producing SCFAs and other acidic metabolites. The accumulation of these metabolites contributed to a reduction in pH value, which played a crucial role in modulating the composition of the gut microbiota and host health [12]. As shown in Figure 5, the initial pH values of the BLK, PFP, and INL were 6.92, 6.83, and 6.85, respectively, with no significant differences among them. The initial pH value of the PFP-80 group was slightly lower than that of the other two groups. This might be caused by the presence of a small amount of glucuronic acid in PFP-80 or by structural changes during fermentation, which led to the release of H^+^ and a decrease in pH. During fermentation, the pH values in the INL and PFP-80 groups were decreased rapidly during the first 12 h, probably due to active microbial metabolism and the production of organic acids [23]. After 48 h of fermentation, the pH value of the BLK group was decreased from 6.92 to 6.52 (ΔpH = 0.4), which might be due to the fermentation of proteins or other non-carbohydrate compounds in the absence of fermentable carbohydrates [24]. In addition, the pH value of the INL group was decreased from 6.85 to 5.51 (ΔpH = 1.34) and remained stable after 12 h. The pH of PFP-80 showed a similar trend to that of the INL group, but it showed a relatively small decrease from 6.83 to 6.14 (ΔpH = 0.69) (*p* < 0.05). This difference might be related to variations in SCFA production during fermentation. Wang et al. [25] reported that some plant-derived polysaccharides could be utilized by gut microbiota to produce SCFAs, leading to a decrease in pH and modulation of microbial composition. A moderate decrease in pH value in the intestine could promote the growth of some helpful microorganisms and inhibit the reproduction of harmful microorganisms [26]. Therefore, the pH value was decreased during the fermentation of PFP, which might affect the proliferation of some specific microbial groups and alter microbial metabolites.

### 3.8. Dynamic Changes in SCFAs

**Figure 6:**
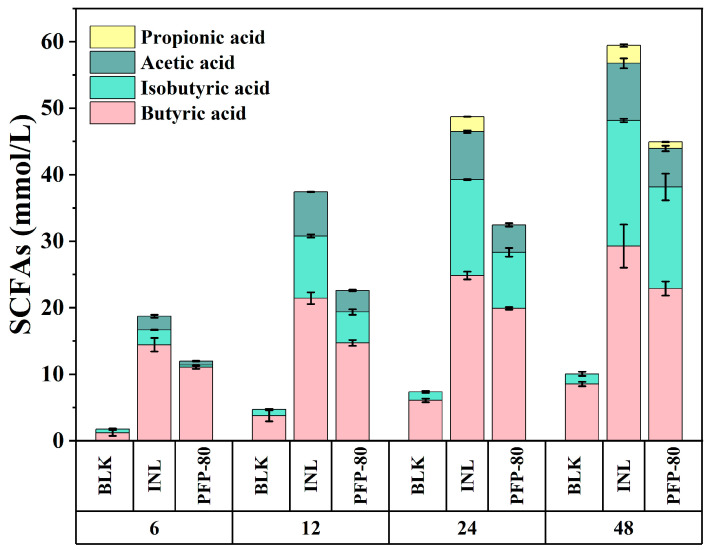
Variations in the content of SCFAs produced at different fermentation time points.

The majority of polysaccharides were indigestible to the upper digestive tract but can reach the colon, where they are fermented by gut microbiota to produce SCFAs. The production of SCFAs was usually considered an indicator of polysaccharide fermentability [27]. The concentrations of total SCFAs in all groups were gradually enhanced with the increase in fermentation time (Figure 6). At all time points excluding 0 h, butyric, acetic, propionic acid, and total SCFA concentrations in the PFP and INL groups were higher than those in the BLK group (*p* < 0.05). After fermentation, the main fermentation products identified were butyric acid, acetic acid, and propionic acid. In the BLK group, the concentrations of SCFAs were increased from 1.74 mmol/L to 10.06 mmol/L, which might be caused by the protein degradation of spoilage bacteria in the intestine [28]. The concentration of total SCFAs in the PFP-80 group was significantly increased to 44.91 mmol/L (*p* < 0.05) after 48 h of fermentation, indicating that PFP-80 could be degraded by gut microbiota to produce SCFAs. After 48 h of fermentation, propionic acid concentration remained at a low level (0.97 mmol/L), while butyric acid and acetic acid in the PFP-80 were increased from 11.06 to 22.89 mmol/L and from 0.59 to 5.79 mmol/L, respectively. Butyric acid acted as a significant energy source for the host and was a major energy source for intestinal epithelial cells. It was widely regarded as a beneficial metabolite due to its immunomodulatory, anti-inflammatory, anti-diabetic, and anti-carcinogenic effects [29,30,31,32]. In addition, ortho- and isovaleric acids were not detected at different fermentation times in all groups, possibly due to the relatively low abundance of specific valeric acid-producing bacteria [33]. Compared with BLK, the total SCFA concentration of PFP-80 was increased approximately four times, which might be associated with the fermentation of PFP-80 by gut microbiota and its contribution to SCFA production [34]. Similar observations have been reported by Cheng et al. [15], who found that total SCFAs in the BLK group were 63.21 mmol/L, while they reached 142.34 mmol/L in the *Polygomatum cyrtonema* polysaccharide (PCP-80%) group, showing an approximately two-fold increase. These results suggested that PFP-80 had a stronger ability to promote SCFA production. It should be noted that this study was conducted using a limited number of donors under controlled in vitro conditions, which might not fully represent in vivo physiological environments. The in vitro fermentation model employed was subject to several inherent limitations. Interactions between gut microbiota and the host immune system were not considered, and neither physiological pH gradients nor SCFA absorption by the intestinal epithelium were simulated. As a result, SCFA accumulation may have been overestimated compared with in vivo conditions. In conclusion, PFP can be degraded into free monosaccharides and subsequently utilized by intestinal microbiota in vitro to produce SCFAs, mainly including butyric acid, acetic acid, and propionic acid. PFP exhibits prebiotic activity and may serve as a potential source of prebiotics, primarily by promoting SCFA production, which contributes to maintaining host health.

### 3.9. Analysis of the Diversity of the Intestinal Microbiota

**Figure 7:**
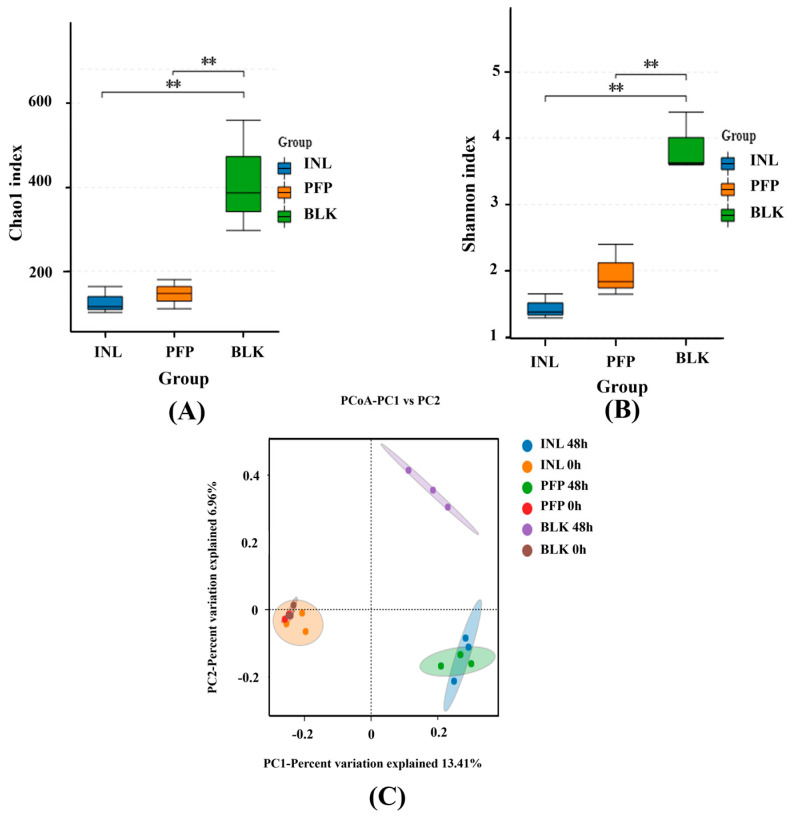
Alpha and beta diversity analysis of intestinal flora of PFP-80 group: (**A**) Chao1 index; (**B**) Shannon index; and (**C**) PcoA. ** indicates *p* < 0.01.

Intestinal microbiota plays an important role in maintaining host health by regulating energy metabolism and preserving the homeostatic balance of the immune system. Polysaccharides are susceptible to decomposition and subsequent utilization by the microbial communities residing in the human intestinal tract, thereby promoting the growth and proliferation of beneficial intestinal bacteria. Therefore, Figure 7A shows the influence of PFP-80 on the human gut microbiota, as assessed by Alpha diversity analysis, which reflects microbial abundance and diversity. As depicted in Figure 7B, both the Chao1 and Shannon indices of the PFP and INL groups were lower compared with the BLK group, which suggests that microbial richness and diversity were reduced. These results suggested that PFP-80 might promote the proliferation of specific microorganisms, and thus reduce the overall diversity and abundance of gut microbiota due to the interspecific competition of dominant microorganisms. This is in agreement with the research of Mao et al. [27], which demonstrated that citrus pectin polysaccharides could significantly reduce gut microbiota diversity. To assess beta diversity, principal coordinate analysis (PCoA) was used to describe the correlation of microbial composition based on OTU levels among the BLK, INL, and PFP-80 groups. PCo1 and PCo2 accounted for 15.75% and 11.20% of the total variance, respectively (Figure 7C). Although the first two axes explained a limited proportion of the variance, PerMANOVA analysis revealed that the differences in microbial community structure among groups were statistically significant (R^2^ = 0.86, Pr (>F) = 0.001). The samples in the INL group and PFP group showed high clustering, and within-group distances were significantly smaller than those between groups. The INL and PFP groups were obviously separated from the BLK group along the first principal component level, suggesting that the gut microbiota exhibited significant differences in their metabolic responses to distinct carbon sources. After 48 h of fermentation, the gut microbiota composition of the PFP and INL groups appeared more similar, while showing significant differences from that of the BLK group.

**Figure 8:**
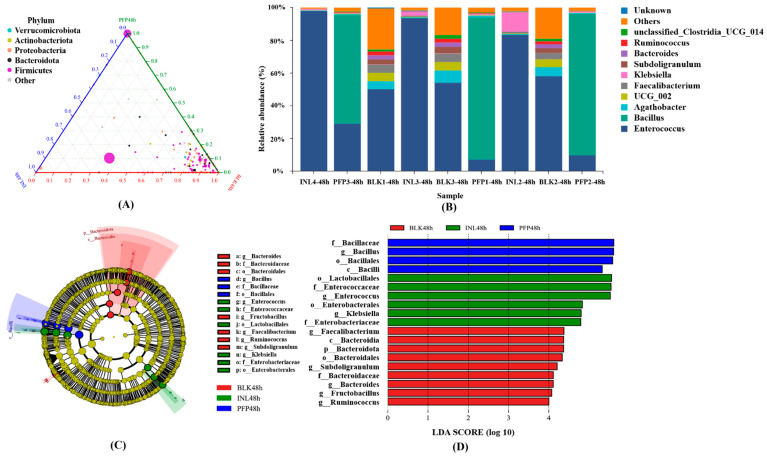
Intergroup comparative analysis of overall gut bacterial composition. (**A**) Ternary phase diagram of species distribution at the gate level. (**B**) Histogram of species distribution at the genus level. (**C**) LDA score histogram. (**D**) Graphlan.

Figure 8A,B show the intestinal microbial community structure at each phylum level after 48 h of fermentation. Actinobacteria, Bacteroidetes, Firmicutes, and Proteobacteria were the predominant phyla in the intestinal habitat, and Bacteroidetes combined with Firmicutes accounted for more than 90% of the total microbiota. In the PFP-80 group, through the analysis of the microbial community structure at the phylum level, it was found that the relative abundance of the Firmicutes phylum significantly increased, accounting for over 55% of the total microbial proportion. By comparison, the PFP-80 group exhibited lower relative abundances of Proteobacteria, Bacteroidetes, and Actinobacteria when contrasted with the BLK group. These results suggested that PFP-80 might not be used for the growth and reproduction of these three bacteria at this stage. Notably, Proteobacteria are the marker bacteria and are often associated with intestinal disorders, causing potential risks to human health. Compared with the INL group, the PFP group showed a higher abundance of Firmicutes, suggesting that PFP-80 could be used as a carbon source for the proliferation of Firmicutes. The Firmicutes are capable of fermenting energy substances in the body’s intestine to produce metabolites such as SCFAs, promoting intestinal health. In addition, the relative abundance of *Bacillus* in the PFP-80 group was increased, while *Ruminococcus gnavus*, *Clostridia UCG-014*, *Subdoligranulum*, and *Klebsiella* were decreased. Although *Klebsiella* showed a decreasing trend, the difference was not statistically significant (*p* > 0.05). *Ruminococcus* has been reported to produce inflammatory polysaccharides that induced dendritic cells to produce inflammatory cytokines such as tumor necrosis factor-α, which led to human inflammatory responses [35]. Wang et al. [36] fed croton to rats and found that it significantly increased the abundance of opportunistic pathogens such as an unnamed genus of *Clostridia UCG-014*, thereby leading to gut microbiota dysbiosis. These results suggested that PFP-80 might inhibit the growth of harmful microorganisms associated with intestinal disorders and metabolic diseases. In conclusion, PFP-80 can have a beneficial effect on human intestinal health by regulating the composition and abundance of intestinal microbial communities.

As shown in Figure 8C,D, the cladogram generated from Linear Discriminant Analysis Effect Size (LEfSe) analysis revealed that four OTUs with high linear discriminant analysis (LDA) scores were significantly enriched in the PFP group. *Bacillus* was identified as the dominant genus in the PFP group, while both *Bacillus* and *Enterococcus* were dominant in the INL group. These key genera contributed substantially to the observed intergroup differences. These findings indicated that ingestion of PFP-80 could significantly modulate gut microbiota composition, which may underpin its beneficial impacts on intestinal health.

## 4. Conclusions

In this study, PFP-80 was isolated from the root of *Polygonatum filipes* using a combination of enzymatic extraction and graded ethanol precipitation. PFP-80 was mainly composed of β-configure fructose and α-configured glucose, with a molar ratio of approximately 97:3. The backbone consisted of →1)-β-D-Fru*f*-(2→, →1,6)-β-D-Fru*f*-(2→ linkages, with branches mainly comprising β-D-Fru*f*-(2→ residues. Furthermore, PFP-80 could effectively modulate gut microbiota composition and promote the production of SCFAs. At the phylum level, an increase in the relative abundance of *Firmicutes* was observed, which is associated with energy metabolism and SCFA production. At the genus level, PFP-80 stimulated the proliferation of beneficial bacteria such as *Bacillus*, while reducing the relative abundance of some potentially harmful taxa, including *Ruminococcus gnavus*, *Clostridia UCG-014* and *Subdoligranulum*. These results suggested that PFP-80 might have a modulatory effect on gut microbial ecology. However, as this study was conducted in vitro with a limited number of donors, further investigations are needed to elucidate the underlying metabolic mechanisms in vivo.

## Figures and Tables

**Table 1:** Chemical component analysis of PFP.

	PFP	PFP-80
Total sugar content (%)	84.14 ± 1.68	94.95 ± 0.34
Uronic acid (%)	3.68 ± 0.20	2.08 ± 0.07
Protein (%)	0.33 ± 0.02	0.32 ± 0.02

**Table 2:** GC-MS data for methylation analysis of PFP-80.

Retention Time (min)	PMAA	Type of Linkages	Relative Ratio (%)	Mass Fragments (*m*/*z*)
7.455	2,5-di-O-acetyl-(2-deuterio)-1,3,4,6-tetra-O-methyl hexitols	Fru*f*-(2→	10.51	87, 101, 102, 129, 161, 162, 205
7.516	2,5-di-O-acetyl-(2-deuterio)-1,3,4,6-tetra-O-methyl hexitols	Fru*f*-(2→	11.36	87, 101, 102, 129, 161, 162, 205
12.4720	1,2,5-tri-O-acetyl-(2-deuterio)-3,4,6-tri-O-methyl hexitols	→1)-Fru*f*-(2→	31.28	87, 101, 102, 129, 162, 189, 233
12.612	1,2,5-tri-O-acetyl-(2-deuterio)-3,4,6-tri-O-methyl hexitols	→1)-Fru*f*-(2→	23.31	87, 101, 129, 45, 161, 190
13.749	1,5,6-tri-O-acetyl-2,3,4-tri-O-methyl glucitol	→6)-Glc*p*-(1→	3.26	87, 99, 102, 118, 129, 162, 189, 233
18.271	1,2,5,6-tetra-O-acetyl-(2-deuterio)-3,4-di-O-methyl hexitols	1,6)-Fru*f*-(2→	7.60	87, 99, 100, 129, 189, 190
18.497	1,2,5,6-tetra-O-acetyl-(2-deuterio)-3,4-di-O-methyl hexitols	1,6)-Fru*f*-(2→	12.69	87, 99, 100, 129, 189, 190

## Data Availability

The original contributions presented in this study are included in the article/Supplementary Materials. Further inquiries can be directed to the corresponding authors.

## Supplementary Materials

The following supporting information can be downloaded at: https://www.mdpi.com/article/10.3390/foods15091561/s1, Figure S1. The GPC of PFP and PFP-80. Figure S2. Monosaccharide composition analysis of PFP-80. Figure S3. FTIR spectra of PFP-80 fractions. Figure S4. The 1H NMR (A),13C NMR (B), COSY (C), NOESY (D), HSQC (E), HMBC (F) spectra analysis of PFP-80. Figure S5. Putative structure of PFP-80. Figure S6. Changes in pH value during fermentation. Figure S7. Variations on the content of SCFAs produced at different fermentation time points. Figure S8. Alpha and Beta diversity analysis of intestinal flora of PFP-80 group. (A) Chao1 index (B) Shannon index (C) PcoA. Figure S9. Intergroup comparative analysis of overall gut bacterial composition (A) Ternary phase diagram of species distribution at the gate level. (B) Histogram of species distribution at genus level. (C) LDA score histogram. (D) Graphlan. Table S1. Chemical component analysis of PFP. Table S2. GC-MS data for methylation analysis of PFP-80. Table S3. Chemical shifts of 1H and 13C for each sugar residue. Table S4. Variations on the content of SCFAs produced at different fermentation time points.
