# Supplementary material for: Structural Characterization and In Vitro Fermentation Properties of Polysaccharides from Polygonatum filipes
Source: Foods. 2026 May 1;15(9):1561. doi: 10.3390/foods15091561 (PMC13164214; doi:10.3390/foods15091561)
Supplement: Supplementary file 1 [file foods-15-01561-s001.zip › foods-4253743-supplementary.pdf]

Structural characterization and in vitro fermentation properties of polysaccharides from *Polygonatum filipes*

Huimin Hu [1,2], Jiawei Wang [1,2], Kaijun Wang [1,2], Ke Chen [1,2], Nike Ding [1,2], Fenghua Wu [1,2], Guanyu Fang [1,2], Xingquan Liu [1,2]， Chaojun Ye [3,*], Peng Wang [1,2,*]

[1] College of Food and Health, Zhejiang Agriculture and Forestry University, Hangzhou 311300, China

[2] National Grain Industry (High-Quality Rice Storage in Temperate and Humid Region) Technology Innovation Center, Zhejiang Agriculture and Forestry University, Hangzhou 311300, China

[3] Key Laboratory of Horticultural Plant Breeding, Wenzhou Vocational College of Science and Technology, Wenzhou Academy of Agricultural Sciences, Wenzhou 325000, China

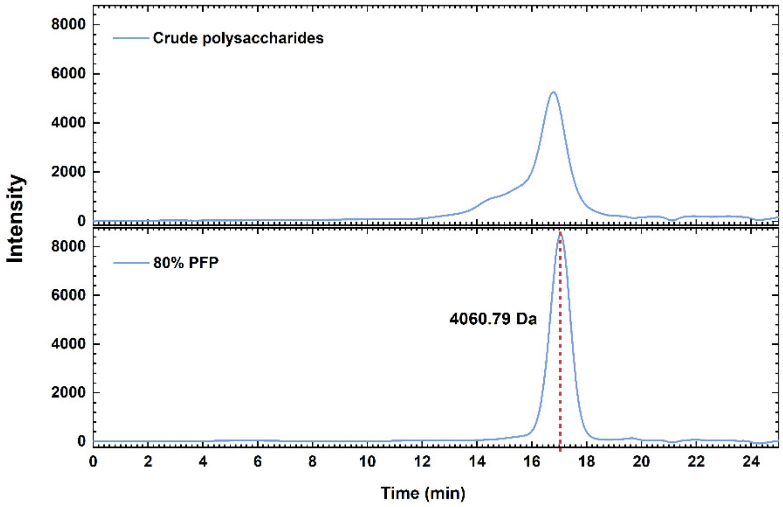

Figure S1. The GPC of PFP and PFP-80

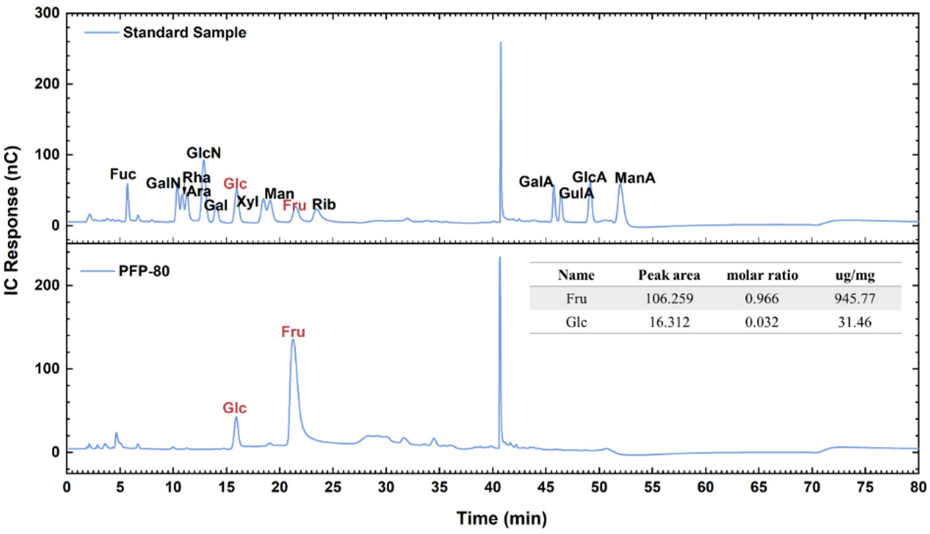

Name	Peak area	molar ratio	ug/mg
Fru	106.259	0.966	945.77
Glc	16.312	0.032	31.46

Figure S2. Monosaccharide composition analysis of PFP-80

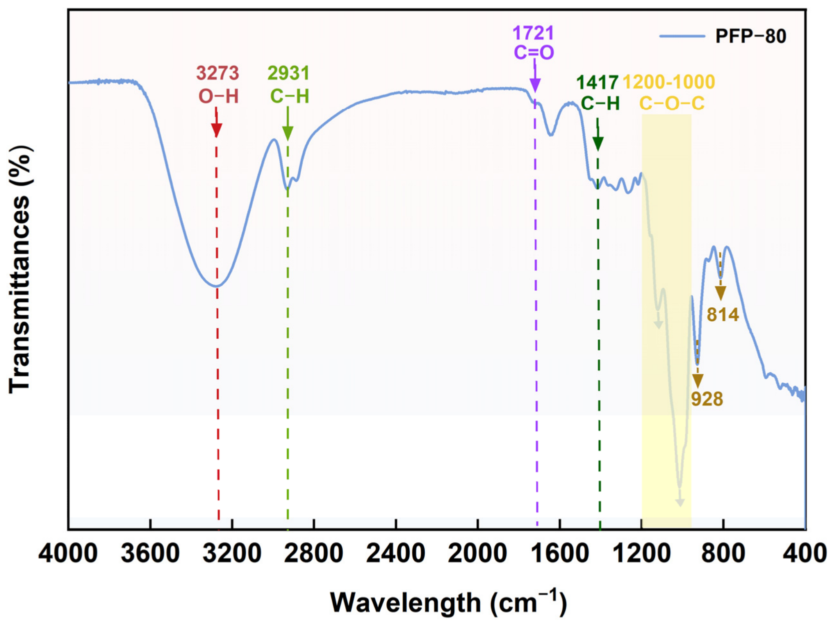

Figure S3. FTIR spectra of PFP-80 fractions

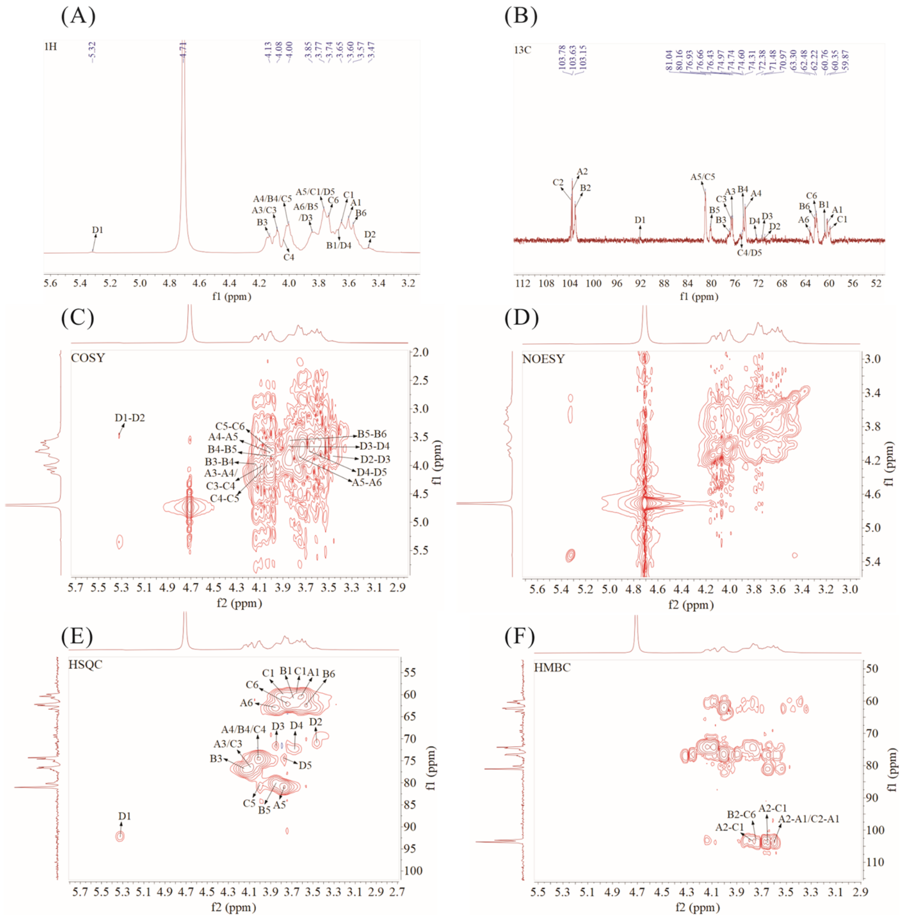

Figure S4. The 1H NMR (A),13C NMR (B), COSY (C), NOESY (D), HSQC (E), HMBC (F) spectra analysis of PFP-80

Figure S4 shows the 1H NMR (A),13C NMR (B), COSY (C), NOESY (D), HSQC (E), HMBC (F) spectra analysis of PFP-80. In Figure S4 (A). No typical anomeric proton signals were observed in the region δ 4.5–5.5, except for δ 5.32, which was consistent with the characteristic feature of fructose residues, as ketoses lack anomeric proton signals. The signal at δ 5.32 was assigned to the anomeric proton of an α-configured glucose residue (residue D), while other proton signals were mainly distributed in the δ 3.1–4.2 region.

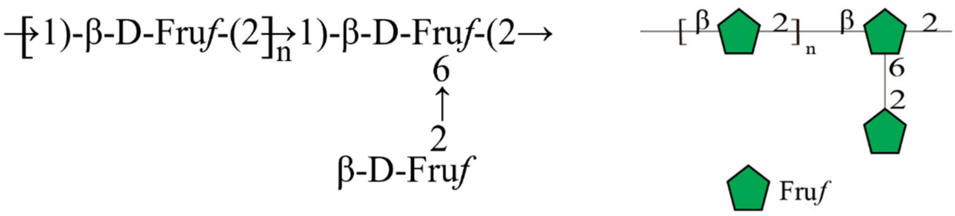

Figure S5. Putative structure of PFP-80.

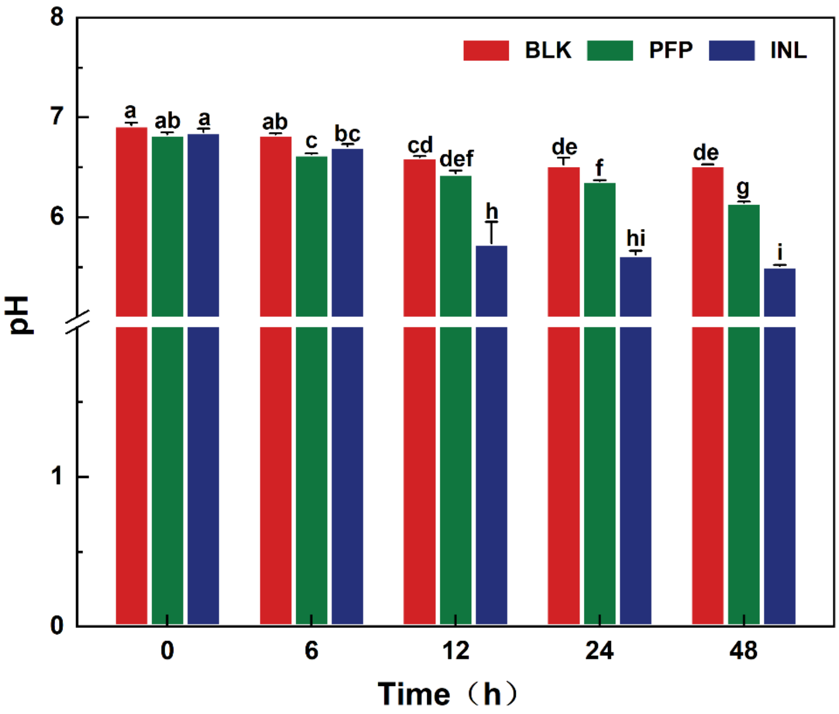

Figure S6. Changes in pH value during fermentation

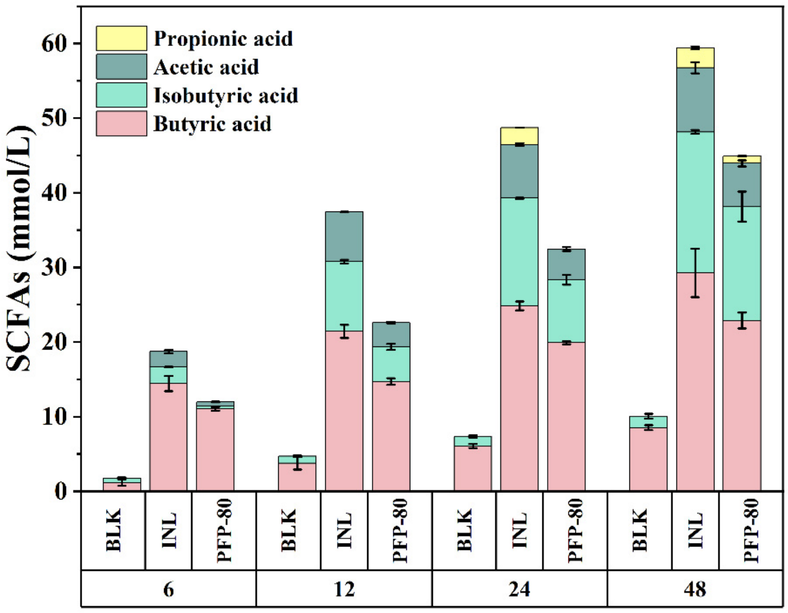

Figure S7. Variations on the content of SCFAs produced at different fermentation time points.

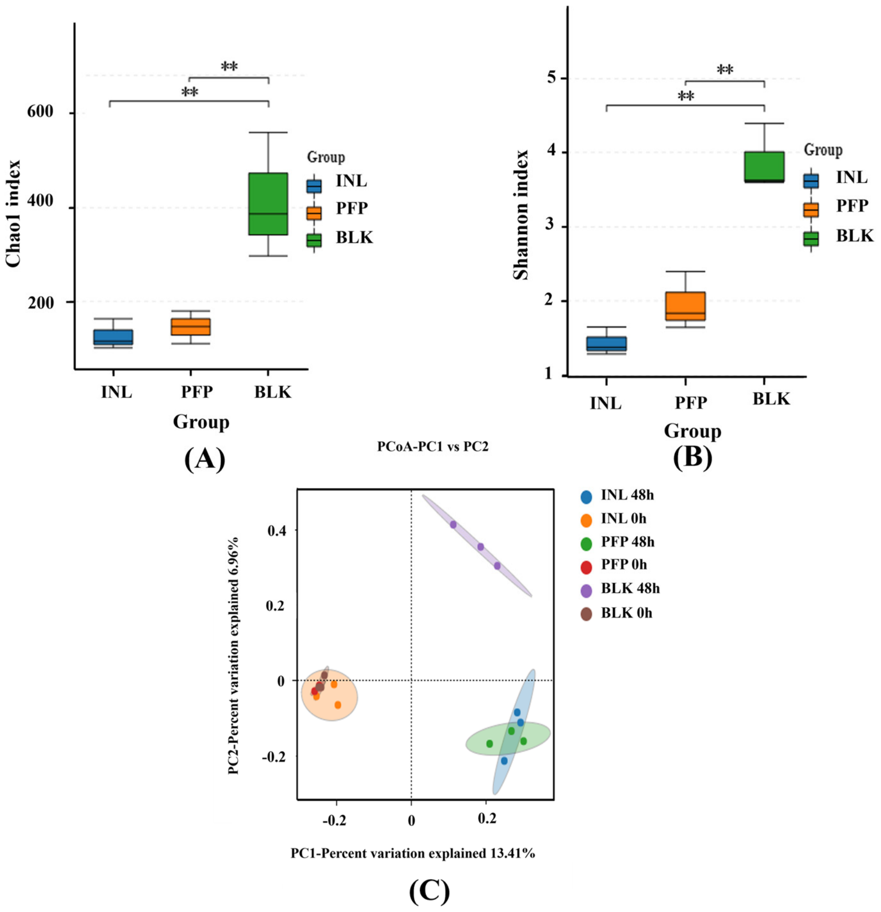

Figure S8. Alpha and Beta diversity analysis of intestinal flora of PFP-80 group. (A) Chao1 index (B) Shannon index (C) PcoA

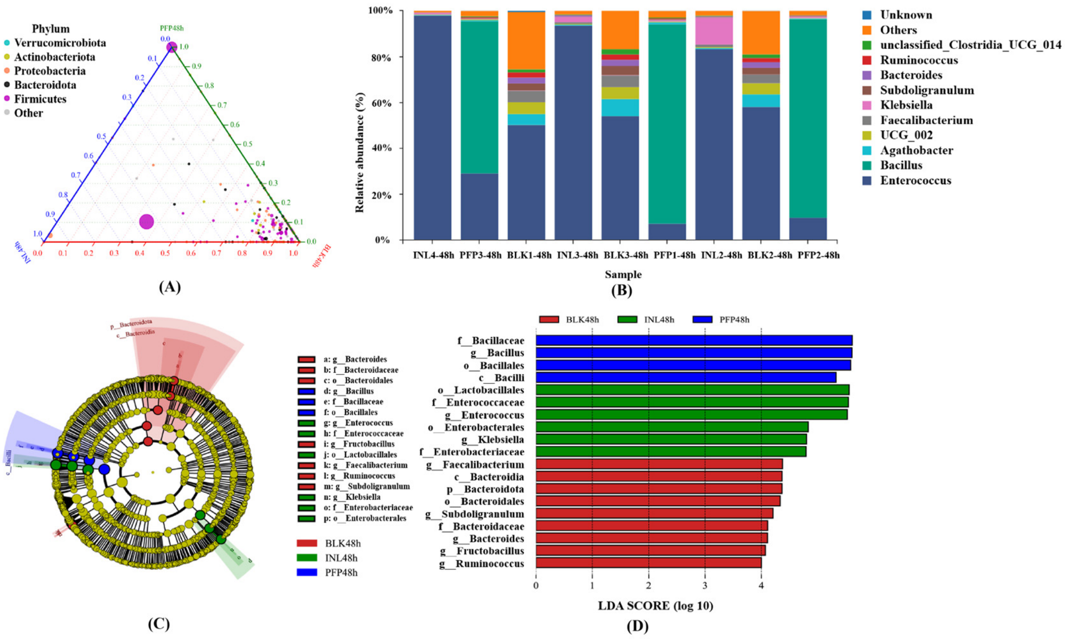

Figure S9. Intergroup comparative analysis of overall gut bacterial composition (A) Ternary phase diagram of species distribution at the gate level. (B) Histogram of species distribution at genus level. (C) LDA score histogram. (D) Graphlan.

Table S1. Chemical component analysis of PFP.

	**PFP**	**PFP-80**
Total sugar content (%)	84.14 ± 1.68	94.95 ± 0.34
Uronic acid (%)	3.68 ± 0.20	2.08 ± 0.07
Protein (%)	0.33 ± 0.02	0.32 ± 0.02

Table S2. GC-MS data for methylation analysis of PFP-80.

Retention time (min)	PMAA	Type of linkages	Relative ratio (%)	Mass fragments (m/z)
7.455	2,5-di-O-acetyl-(2-deuterio)-1,3,4,6-tetra-O-methyl hexitols	Fru*f*-(2→	10.51	87, 101, 102, 129, 161, 162, 205
7.516	2,5-di-O-acetyl-(2-deuterio)-1,3,4,6-tetra-O-methyl hexitols	Fru*f*-(2→	11.36	87, 101, 102, 129, 161, 162, 205
12.4720	1,2,5-tri-O-acetyl-(2-deuterio)-3,4,6-tri-O-methyl hexitols	→1)-Fru*f*-(2→	31.28	87, 101, 102, 129, 162, 189, 233
12.612	1,2,5-tri-O-acetyl-(2-deuterio)-3,4,6-tri-O-methyl hexitols	→1)-Fru*f*-(2→	23.31	87, 101, 129, 45, 161, 190
13.749	1,5,6-tri-O-acetyl-2,3,4-tri-O-methyl glucitol	→6)-Glc*p*-(1→	3.26	87, 99, 102, 118, 129, 162, 189, 233
18.271	1,2,5,6-tetra-O-acetyl-(2-deuterio)-3,4-di-O-methyl hexitols	1,6)-Fru*f*-(2→	7.60	87, 99, 100, 129, 189, 190
18.497	1,2,5,6-tetra-O-acetyl-(2-deuterio)-3,4-di-O-methyl hexitols	1,6)-Fru*f*-(2→	12.69	87, 99, 100, 129, 189, 190

Table S3. Chemical shifts of 1H and 13C for each sugar residue.

Code	Glycosyl residues	Chemical shifts(ppm)					
		H1/C1	H2/C2	H3/C3	H4/C4	H5/C5	H6/C6
A	→1)-β-D-Fru*f*-(2→	3.60	/	4.09	4.01	3.77	3.85
		60.35	103.63	76.43	74.31	81.04	63.3
B	β-D-Fru*f*-(2→	3.67	/	4.14	3.98	3.84	3.57
		60.76	103.15	76.93	74.6	80.16	62.48
C	→1,6)-β-D-Fru*f*-(2→	3.77,3.65	/	4.1	4.04	4	3.74
		59.87	103.78	76.66	74.97	81.13	62.22
D	→6)-α-D-Glc*p*-(1→	5.32	3.46	3.85	3.67	3.77	n.d
		92.19	70.97	71.48	72.38	74.97	n.d

Table S3 shows the chemical shifts of 1H and 13C for each sugar residue. By analyzing the cross peaks in the anomeric region of the ^{1}H NMR and HSQC spectra, the anomeric signal of sugar residue D was identified at δH-1 5.32/δC-1 92.19, suggesting that it was an α-configured glucose residue. In addition, in the COSY spectrum, four cross-peak signals were found, which were δH 5.32/δH 3.46, δH 3.46/δH 3.85, δH 3.85/δH 3.67 and δH 3.67/δH 3.77 of sugar residue D, respectively. Then, the chemical shifts of the carbon of the sugar residue D were attributed by the HSQC spectra, with chemical shifts at δC-1 92.19, δC-2 70.97, δC-3 71.48, δC-4 72.38 and δC-5 74.97 of sugar residue D, which predicted that the sugar residue D may be →6)-α-D-Glc*p*-(1→.

Table S4. Variations on the content of SCFAs produced at different fermentation time points.

Samples	Time (h)	SCFAs (mmol/L)				
		butyric acid	isobutyric acid	acetic acid	propionic acid	total
BLK	0	ND	ND	ND	ND	ND
	6	1.18±0.43h	0.56±0.13ef	ND	ND	1.74±0.32h
	12	3.78±0.85gh	0.92±0.11ef	ND	ND	4.7±0.68gh
	24	6.08±0.27fg	1.27±0.16ef	ND	ND	7.35±0.31fg
	48	8.53±0.34ef	1.53±0.32ef	ND	ND	10.06±0.21f
INL	0	ND	ND	ND	ND	ND
	6	14.43±1.02d	2.25±0.03e	2.04±0.22e	ND	18.72±1.19e
	12	21.43±0.88c	9.36±0.24c	6.63±0.02bc	ND	37.42±1.01c
	24	24.85±0.59b	14.41±0.08b	7.21±0.18b	2.25±0.01b	48.72±0.90b
	48	29.26±3.26a	18.89±0.25a	8.60±0.74a	2.66±0.20a	59.41±3.93a
PFP-80	0	ND	ND	ND	ND	ND
	6	11.06±0.24e	0.32±0.02f	0.59±0.04f	ND	11.97±0.68f
	12	14.71±0.45d	4.65±0.41d	3.23±0.11d	ND	22.59±0.90e
	24	19.90±0.21c	8.43±0.65c	4.12±0.26d	ND	32.45±1.03d
	48	22.89±1.06bc	15.26±2.01b	5.79±0.41c	0.97±0.03c	44.91±3.52b

ND means undetected or lower than the detection limit of the method.

Different letters in the figures indicate statistically significant differences between groups.
